# Role Value, Occupational Balance, and Quality of Life: A Cross-Sectional Study on Exploring the Urban Older People Perspective in South Korea

**DOI:** 10.3390/ijerph19053054

**Published:** 2022-03-05

**Authors:** Myoung-Ok Park, Ji-Hyun Lee

**Affiliations:** 1 Department of Occupational Therapy, Division of Health Science, Baekseok University, 1, Baekseokdaehak-ro, Dongnam-gu, Cheonan-si 31065, Chungcheongnam-do, Korea; 2 Department of Physical Therapy, Division of Health Science, Baekseok University, 1, Baekseokdaehak-ro, Dongnam-gu, Cheonan-si 31065, Chungcheongnam-do, Korea; jihyun.lee@bu.ac.kr

**Keywords:** older adults, occupational balance, role value, quality of life

## Abstract

The purpose of this study was to investigate the role value, occupational balance, and quality of life among urban older adults in South Korea. We recruited 90 urban older adults in Seoul, Gyeonggi-do and Chungcheong-do. Assessments used (1) Role Checklist, (2) Life Balance Inventory (LBI), and (3) WHO Quality of Life Scale abbreviated version (WHOQOL-BREF). Our results showed that the roles that were perceived as very valuable were as family members, housekeepers, and guardians (in descending order). The roles that were perceived as less valuable were students, volunteers, and organizational members (in descending order). The activities that individuals were actively pursuing were hygiene management, rest, and healthy eating (in descending order). By contrast, the activities that were not being actively pursued were composing (music, poetry), preparing for event planning, dancing, yoga, and taekwondo. The total score of the Role Checklist and WHOQOL-BREF total (r = 0.343, *p* < 0.01), K-LBI total and WHOQOL-BREF total (r = 0.386, *p* < 0.01), and role value total and K-LBI (r = 323, *p* < 0.01) showed a statistically significant correlation. As a result of the regression analysis, the sub-item of work balance that affected the quality of life was managing appearance (R^2^ = 51.7, *p* < 0.001). These data showed that the role of urban older adults in Korea was mainly played within the family. The level of participation was low in the areas of instrumental daily life activities, work, leisure, and social participation. We propose that this population needs to be provided with opportunities for active aging through broader professional participation.

## 1. Introduction

Population aging is a global social issue worldwide due to low fertility rates. This is due to the revitalization of economic activity in females and the extension of life expectancy due to the development of medical technology. An aging society, as defined by the United Nations, refers to when the older adults aged above 65 years exceeds 14% of the total population. A super-aging society refers to when the older adults aged above 65 years exceeds 20% of the total population. According to the Korea National Statistical Office [1], the older adults in Korea are continuously increasing by 0.32% to 0.68% per year and Korea is becoming a super-aging society. In contrast to the steady increase in the older adults, the economic and social status of the older adults has decreased due to changes in the industrial structure and workforce demands over time [2]. In addition, conditions have lowered the quality of life of the older adults, such as a narrowing of their position as a member of society, changes in roles and functions within the family, and disabilities in the living environment [3]. Gill et al. have reported that the role of human beings changes as they live, and their value changes over each life stage [4]. Role value refers to the importance given to each of the roles an individual undertakes in his or her life.

According to role theory, humans perceive themselves as social beings while performing roles and social roles are connected with age or life stage [5]. Old age is viewed as a stage of life in which a role previously performed is lost rather than acquiring a new role, which not only loses social roles but also affects occupational balance in daily life [6].

Occupational balance refers to a state in which one is participating in and maintaining a balance in one’s daily activities to maintain a healthy life and well-being [6]. Occupational balance is divided into basic daily life activities, instrumental daily life activities, rest and sleep, education, work, play, leisure, and social participation. Therefore, any meaningful activity in which human beings participate while leading a life can be defined in this way [7]. The concept of occupational balance may be complex and different; however, it is related to health and well-being satisfaction [8,9]. Christiansen [10] has defined occupational balance as the relationship between the use of time and activity patterns, biological rhythms of time, and life activities. He argues that it is related to life satisfaction [10]. A state in which time use is properly allocated to productive activities, leisure time, and self-management is defined as a good occupational balance state [11]. Hence, a well-balanced life is achieved by properly distributing psychological and physical energy, while aligning lifetime with oneself and properly dividing and using occupation areas [12]. Ramos et al. [13] have reported that occupational balance is related to quality of life. Additionally, a study on occupational balance and health has shown that a greater awareness of life balance is correlated with a better health status and quality of life [13]. Quality of life reflects the interaction between social conditions and members of society. It seeks to quantify the feeling of satisfaction that makes an individual’s life valuable and rich [13,14]. Quality of life reflects many variables, such as work, leisure, economy, living environment, stability, family relationship, social relationship, and religion [14]. Individual roles and occupational participation changes over time, which can affect quality of life [15].

The older adults experience changes with their differing roles and occupational balance over time. This is due to physical, mental, and environmental changes in their lives [16].

When social roles change due to changes in life, such as bereavement or retirement, changes in occupational participation also occur [17]. Older people often have less diversity in their daily life as they get older, and they spend more time in passive leisure activities [18]. For successful aging, when an individual participates in various tasks, a harmonious balance must be achieved in the participation [19]. For the older adults over 65 years of age, occupational-balanced life is the basis for protecting their health and living a well-being [18,19]. According to a previous study, it was reported that, in the case of the older adults in rural Korea, the family role fell sharply, while the health and self-management roles were high, but the social role did not change significantly [20]. Additionally, in terms of quality of life and life satisfaction, it was found that the higher the monthly cost of living or the higher the educational background, the higher the income from the farm family, and the higher the level of life satisfaction [21].

This study aimed to identify the current role, occupational balance, and quality of life for urban older adults in South Korea, and to investigate their relevance. The following research questions guided the study: (a) What are the main roles that the urban older adults valued in South Korea?; (b) Is occupation balanced by time use?; (c) The relationship between role value, occupational balance, and quality of life, what are the factors of role value and occupational balance that affect quality of life?

## 2. Materials and Methods

### 2.1. Study Design and Participants

This study is a cross-sectional research study. Data were collected for approximately two months, from 2 April 2019 to 9 June 2019. The estimation of the sample size required for this study was calculated with the G*Power software, version 3.1.9.7 (Heinrich-Heine-University software, Düsseldorf, Germany). The sample size was measured based on the regression model analysis to analyze the occupational balance factors that affect the quality of life, the final purpose of the study. The regression model was selected based on linear multiple regression analysis by selecting the median effect size (Effect size f2 = 0.15), alpha = 0.05, power = 0.095, and 5 minimum predictors among K-LBI sub-activities. According to this analysis, the minimum sample size should include a minimum of 95 participants.

This included individuals up to the age of 60 years. Individuals were recruited in front of ward offices, churches, home visits, and older adult welfare centers in the urban areas of Seoul, Gyeonggi, and Chungcheong. The criteria for including participants were: (a) senior citizens aged 60 or older in urban areas of cities, counties, and districts; (b) no problem with audiovisual comprehension; (c) can participate in the survey by themselves and their guardians; and (c) voluntarily agreed to participate in the study. Even if they live in the suburbs of the city, the older adults aged 60 or older belonging to the administrative districts of Eup, Myeon, and Ri were excluded. Therefore, ninety-five people were initially recruited; however, 90 were included in the analysis because 5 individuals could not fully participate in the analysis due to individual circumstances.

### 2.2. Main Measures

#### 2.2.1. Role Value

Role value of participants was measured with Role Checklist (RC). The Role Checklist, created by Oakley [22], was applied to evaluate role value levels. This tool evaluates the past, present, and future roles for the 10 most common roles, such as student, workplace, guardian, friend, and religious person. The value level that the participant feels about each role is recorded. In this study, the value of the role was evaluated and applied as an indicator of how each participant felt about this role. Previous studies have shown a test-retest reliability (Kappa value) of 0.74–1.00.

#### 2.2.2. Occupational Balance

Occupational balance was measured with the Life Balance Inventory (LBI) [23]. The LBI developed to investigate the balance and imbalance of tasks that compose life, is based on the life balance model. The LBI includes 53 activities (e.g., personal hygiene, managing appearance, relaxing, meeting new people, cooking, and eating out). It is divided into four subscales: health, relationship, identity, and challenge. It is measured based on subjective experience. The life balance scale of the older adults is divided into Stages 1 and 2. In Stage 1, participants answer “yes” if they are performing this activity or want to perform it. In Stage 2, for each activity that was answered with “yes”, participants respond whether time spent performing this activity in the last month was sufficient. Therefore, the scoring system is as follows: 1 or 0 points for answering “yes” or “no”, respectively, at Stage 1. At Stage 2, a 5-point Likert scale is used. If the sufficiency of time spent performing the activity was “always insufficient”, 1 point is awarded. If time spent is ”sometimes insufficient”, 2 points are awarded. If time spent “appears to be appropriate for me”, then 3 points are awarded. If time spent is “sometimes a lot”, then 4 points are awarded. Finally, if time spent is “always sufficient”, then 5 points are awarded. The LBI has demonstrated acceptable internal consistency and content validity as a measure for life balance, with Cronbach’s alpha = 0.89–0.97 [23]. In this study, the Korean version of the LBI (K-LBI), which was verified for reliability validity by Park et al., was applied [24]. The reliability of K-LBI in the older population is Cronbach’s alpha value of 0.875.

#### 2.2.3. Quality of Life (QoL)

QoL was measured with the WHO Quality of Life Scale, Bref version. The WHO Quality of Life Scale (WHOQOL-BREF) is an abbreviated version of WHOQOL-100. This is used to evaluate the quality of life of urban older adults [25]. It has 26 items and 4 subscales: (a) physical health, (b) psychological, (c) social relationships, and (d) environment. This assessment includes sub-items that ask: “how many things have you experienced during the past 2 weeks”, “how much have you experienced certain things”, and “how satisfied and good are in various areas of your life”. Using Likert’s 5-point scale, scores are marked as follows: “Very dissatisfied”, “Not at all” = 1 point; “Dissatisfied”, “Slightly agree” = 2 points; “Yes”, “Neither satisfied nor dissatisfied” = 3 points; “Satisfied”, “Mostly agree” = 4 points; and “Totally agree”, “Very satisfied” = 5 points. The correlation with test-retest reliability was significant in all item scores of the Korean version of WHOQOL and WHOQOL-BREF (*p* < 0.0001). The Cronbach’s alpha = 0.898. The value of each domain was also high (0.583–0.777), and the internal consistency was good [25].

### 2.3. Procedures

The main measures were included in a study questionnaire. This questionnaire was administered to the older adults who consented to participate in the study. For the recruitment of research participants, official notices about research participants were delivered through community welfare institutions, churches, the heads of leisure facilities for the older adults, and local community centers. Research participants were recruitment from organizations that agreed to the official document. Although it is a self-report-type test based on a questionnaire, two occupational therapists participated in this process for explanation of the study and questionnaire type evaluation. Prior to examination and explanation, occupational therapists received the same training from the study director to minimize inter-rater errors. The purpose, procedure, and confidentiality of this study were explained to the participants and legal guardians. Participation of older adults was voluntary, anonymous, and secret. All were advised that there was no penalty for leaving the study at any point during the research process, and that the participant may withdraw from the study at any time if he/she does want to. Additionally, participants were informed that their data would not be used for any purpose other than the research. Only participants or legal guardians who provided written informed consent were included in the study analysis. All of these processes were conducted in accordance with the ethical principles for research with human subjects of the Declaration of Helsinki. As it was a survey-type test targeting the older adults, it took about an hour. During the investigation process, it was explained that sufficient rest can be recommended or stopped for the older adults who have difficulty concentrating or sitting due to deterioration in physical condition. Fortunately, no potential hazard occurred during the course of the investigation. All data collection for all participants and anonymity were ensured for subsequent subdivisions.

### 2.4. Data Analysis

The statistical analysis was performed using SPSS 20.0 version (IBM, New York, NY, USA). We analyzed the data using a two-tailed test at significance levels of 0.05. Descriptive analysis was used of general characteristics, role value, and occupational balance. Covariance analysis was performed excluding the effect of role value to find out the difference in quality of life by level of occupational balance of participants. The correlation between the participants’ role value, occupational balance, and quality of life was analyzed using Pearson’s correlation coefficient. Stepwise multiple regression analysis was performed to investigate the effect of role value and occupational balance on quality of life.

## 3. Results

### 3.1. Participant Characteristics

The participant characteristics are shown in Table 1 below. The participants’ mean age was 68.03 years. Additionally, there were more females (64.4%) than males (35.6%). The highest level of educational attainment was high school (33.3%), followed by college (22.2%), elementary school (21.1%), uneducated (3.3%), and graduate school (2.2%).

### 3.2. Current Role Values, Occupational Balance, and Quality of Life

#### 3.2.1. Role Value Rate of Participants

The types of role values are presented in 10 categories: students, office workers, guardians, housework, friends, family members, religious people, hobbyists, and organizational members. As a result of identifying the current role value level of older adults, the items that were identified as very valuable for participants were family member (54.4%), housekeeper (45.6%), and guardian (40.0%).

**Figure 1:**
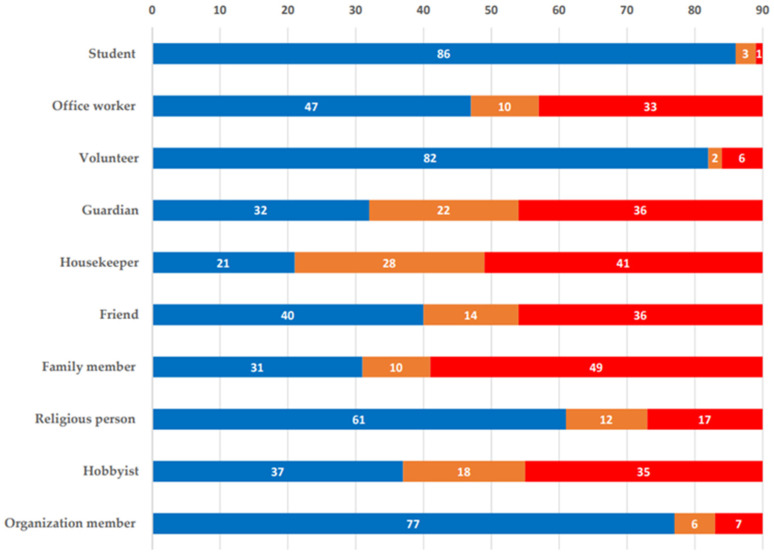
Role value rate of participants.

By contrast, the roles that were reported to have no value included student (95.6%), volunteer (91.1%), organization member (85.6%), religious person (67.8%), office worker (52.2%), friend (44.4%), and hobbyist (41.1%) (Figure 1).

#### 3.2.2. Perceived Appropriateness of Occupation Balance and Use of Time of Participants

K-LBI was used to investigate the proportion of urban older adults who underwent different activities and the level of time used for each activity. This was used to assess occupational balance. Among the 53 activities measured, activities in which >90% of the older adults participated were hygiene (97.8%), relaxing (95.6%), eating nutritiously (94.4%), doing things with family (93.3%), and getting adequate sleep (92.2%) (Appendix A).

The results of investigating the appropriateness of time spent for each activity among urban older adults’ current activities through K-LBI were as follows. Activities in which >60% of participants reported using their time appropriately were hygiene (71.1%), eating nutritiously (70.0%), relaxing (64.4%), using public transport (63.3%), and watching TV (61.1%) (Appendix B).

#### 3.2.3. Role Value Score, Occupational Balance, and Quality of Life of Participants

Role value total score of participants was 7.38 ± 3.75. There was no significant difference between men and women older adults. The score of the participant’s occupational balance was checked through K-LBI. As a result, the average total score of K-LBI was 72.74 ± 28.66, and there was a statistically significant difference between male and female older adults (t = 24.078, *p* < 0.01). Among the sub-items of K-LBI, the identity items scored an average of 25.77 ± 1.96, indicating a statistically significant difference between men and women (t = −3.086, *p* < 0.05). Quality of life was examined using the WHOQOL-BREF score. The overall mean score was 86.06 ± 2.947. The quality of life for each sub-domain was 20.56 ± 0.89 in the physical health area, 15.42 ± 2.47 in the psychological area, 9.64 ± 1.73 points in the social area, and 29.13 ± 4.76 points in the environmental area. There was no difference in QoL sub-items between men and women older adults (Table 2).

#### 3.2.4. Results of Covariance Analysis on Corrected Quality of Life According to Occupational Balance

The K-LBI score was analyzed categorically, and the quality of life according to occupational balance was analyzed. As for the co-variate factors, the total score of role values, which showed relevance in the correlation analysis, was selected as the control factor. As a result, there was no significant difference in the corrected quality of life according to the level of occupational balance (Table 3).

#### 3.2.5. Relationship between Role Value, Occupational Balance, and Quality of Life

As a result of identifying the linear co-relationship between role value, occupational balance, and quality of life, the total score of role value and WHOQOL-BREF total (r = 0.343, *p* < 0.01), K-LBI total and WHOQOL-BREF total (r = 0.386, *p* < 0.01), and role value total and K-LBI (r = 323, *p* < 0.01) showed a statistically significant correlation (Table 4).

The correlation between the K-LBI sub-items and the WHOQOL-BREF sub-items of participants was identified. Physical health of quality of life showed a weak correlation in the identity domain (r = 0.258, *p* < 0.01) and challenge and interest domain (r = 0.225, *p* < 0.05). The psychological domain of quality of life showed a moderate correlation (r = 0.400, *p* < 0.05). The social relation domains of quality of life were health (r = 0.397, *p* < 0.01), relationship (r = 0.461, *p* < 0.01), identity (r = 0.397, *p* < 0.01), challenge and interest (r = 0.422, *p* < 0.01) and showed a correlation with the activities. The environmental domains of quality of life were health (r = 0.322, *p* < 0.01), relationship (r = 0.353, *p* < 0.01), identity (r = 0.318, *p* < 0.01), challenge and interest (r = 0.355, *p* < 0.01) and showed a correlation (Table 4).

#### 3.2.6. Effect of Occupational Balance and Role Value on Quality of Life

Through a stepwise multiple regression analysis, the factors of occupational balance and role value that affect the quality of life of participants were identified. Briefly, we used the total WHOQOL-BREF score as the dependent variable. The items that answered ‘yes’ in the K-LBI evaluation step 1 and the role value total score were applied as independent variables. As a result of this analysis, the regression formula of Y(QOL) = 66.952 + 2.645 * (appearance management) was revealed. Each coefficient was significant (R^2^ = 0.517, *p* < 0.001, Table 5).

## 4. Discussion

In this study, the relationship between role value, occupational balance, and quality of life in the urban older adults in Korea was investigated. Role value was investigated using the Role Checklist. The roles that participants currently felt were valuable were (in descending order of value) as family members, housekeepers, guardians, friends, hobbies, office workers, religious people, organization members, volunteers, and students. Hong and Lee [26] have studied Korean adults and shown that the amount of time used in the occupation area for each life stage depends on the role played by the individual in each life stage. According to their study, adolescents spent more time as students. Moreover, in this life stage, paid activities and social participation are more valued. On the other hand, it was reported that the role in the family is the most important in old age [27]. Old age has a different life pattern than that of young adulthood due to a loss of a defined role and leads to changes in value levels [28]. This study identified the occupational balance of the urban older adults who participated in this study using the K-LBI. The activities that were most time-occupying were hygiene management, rest, healthy eating, spending time with family, and getting adequate sleep. These tasks were related to basic daily life and housework when compared with most other activities. By contrast, a previous study has shown that young adults in their 20s and 40s respond that they are currently most engaged in activities such as internet access, followed rest, eating out, getting adequate sleep, and family [29].

Compared with previous studies, the item that showed a significant difference in the activities of the urban older adults who participated in this study was the use of the internet. This is a result that indicates that even the older adults in urban environments do not participate in activities by using the internet as a means of social communication as much as young adults. The previous study carried out by Yang and Seol [29] has shown that the activities that young adults report little or no participation are writing lyrics, composing, embroidering/sewing, group exercise, gardening/gardening, and mentoring. In contrast, the activities that the urban elderly in this study reported little or no participation were songwriting, composition, event planning, dance, yoga, taekwondo, performing arts, and storytelling.

This suggests that young adults engage less in static hobbies or quiet activities, whereas the older adults participate in fewer creative activities requiring higher-order cognitive thinking [30,31]. These results clearly show a difference between older- and younger-aged individuals in activities.

The items that older adults reported having appropriate time to participate were hygiene management, eating healthy, resting, using public transportation, watching TV, getting adequate sleep, traditional rituals, holiday celebrations, appearance management, health care, and spending time with family. In addition, the items with inappropriate time use were traveling, spending time with family, spending time in nature, spending time with friends, getting adequate sleep, doing housework, resting, and taking care of health, spending time with a spouse/lover, and watching movie/theater/ sports game.

According to a study on the older adults in Sweden [32], occupational balance was investigated for older workers 65 years of age or older. As a result, it was reported that individual abilities, utilization of community resources, harmonious harmony with one’s occupation, and personal values and unity contribute to achieving a good occupational balance. Therefore, it is necessary to recommend the older adult to find meaning through social activities rather than spending their time only on activities at home such as hygiene management, rest, and healthy eating.

Although there was no difference in the role value and overall quality of life score between the male and female older adults who participated in the study, there was a significant difference in the overall score of work balance and the difference between the male and female in the identity part among the subcategories. Although the conditions were not the same as in this study, the previous study showed that there was a difference in occupational balance between men and women in a nursing home [33]. The result is similar in this study.

After controlling the role value, which showed a weak correlation with the quality of life, the statistical significance of the corrected quality of life according to the occupational balance level was investigated. As a result, there was no difference in the quality of life according to the level of occupational balance while controlling the role value of the urban older adults who participated in this study. This is considered to be because the Korean urban older adults who participated in this study did not participate in various activities.

The items of K-LBI were analyzed to activities of daily living, instrumental activities of daily living, health care, rest and sleep, work, play, leisure, and social participation, which are eight occupation areas proposed by the Occupational Therapy Practice Framework (OTPF) [34]. For the older adults who participated in this study, rest and sleep activities of daily life showed the most occupational participation. Work education, leisure, and social participation showed occupational deprivation.

For the older adults, activities in daily life are suppressed due to physical illness and a decline in economic income from retirement [35]. Previous studies have reported that daily life activities for personal maintenance increase in the older adults. Moreover, the lifetime related to paid activities decreases with old age [35]. The WHO [36] has noted the need for processes to optimize health, participation, and security opportunities that improve quality of life as people age. This is referred to active aging; the importance of this is not only the ability to be physically active or to participate in the workforce, but to be able to retain social, economic, cultural, mental, and civic problems responsibilities [37]. Therefore, community-based activity participation services should be expanded to support the participation of the older adults in various occupational therapy activities.

There was a weak correlation between the participants’ role value, quality of life, and total score of occupational balance. However, it showed meaningful results among the subcategories of occupational balance and quality of life. The psychological area of quality of life showed a moderate correlation with relational activities during daily work, and the social-relational area of the quality of life showed a moderate correlation with relational activity, challenge, and performance of interesting activities. A previous study targeting the older adults in rural South Korea reported that social activities and hobbies were positive factors influencing life satisfaction [37,38]. In this study, it was found that relationship activities are related factors that have a close influence on the quality of life of the urban older adults. Another previous study has also shown that leisure activities account for a large proportion of the social connections of older adults. Volunteering, cultural activities, vacations, sports, reading, hobbies, and shopping were successful predictors of social connectivity in the older adults. On the other hand, it was reported that passive activities such as watching TV, using computer, and listening radio were not related to social connectivity [39]. Considering these points, it is considered that life care that encourages active social activities to improve the quality of life is necessary.

In stepwise multiple regression analysis, it was found that the better the appearance management, the higher the quality of life. A previous study has reported that the degree of taking care of appearance in the older adults affects the quality of life [40]. A study on the older adults in Japan also showed that internet use and taking care of appearance gives meaning to the life of the older adults and contributes to improvements in the quality of life [41]. These points support the results of this study. Older people may feel a decrease in self-esteem due to age-related physical changes [42]; therefore, it is necessary to actively provide training for taking care of appearance to improve their quality of life. This study has some limitations. The study only explored the relationship between role value, occupational balance, and quality of life for urban older adults in South Korea. It did not clearly suggest the differences with urban older adults in Western countries. In future studies, comparative studies with other countries should be conducted.

## 5. Conclusions

A balance of time use in role and occupation is very important for active aging. For the older adults who participated in this study, quality of life in daily life was related to occupational balance; however, the work of the older adults was mainly distributed in basic daily activities, such as rest and sleep. In addition, taking care of one’s appearance contributed to improving quality of life. This suggests that education and training are necessary to promote occupational balance via activities that expand social participation and improve the quality of life in older adults.

## Figures and Tables

**Table 1:** Characteristics of participants (N = 90).

Characteristics			All	N (%)
Age (Year)	60–69		63.91 ± 3.03	
	70–79		73.50 ± 2.98	
	≥80		84.17 ± 2.92	
	Mean ± SD		68.03 ± 6.80	
	Min.–Max.		60–88	
Gender		Male Female		32 (35.4) 58 (64.4)
Education		Uneducated Elementary school Middle school High school College Graduate		3 (3.3) 19 (21.1) 16 (17.8) 30 (33.3) 20 (22.20) 2 (2.2)
Employed		No Yes		38 (42.2) 52 (57.8)
Marital status		Married Single Widowed Divorced		72 (80.0) 14 (15.6) 2 (2.2) 2 (2.2)
Health status		Very good Good Average Bad Very bad		26 (28.9) 5 (5.6) 38 (42.2) 18 (20.0) 3 (3.3)
Financial preparation for retirement		Prepared Very well prepared Average No ready at all Not prepared		40 (44.4) 7 (7.8) 36 (40.0) 5 (5.6) 2 (2.2)
Religion		Catholic Buddhism Christian No religion		10 (11.1) 15 (16.7) 23 (25.6) 42 (46.7)

**Table 2:** Characteristics of participants among occupational balance, quality of life, and role value total score (*n* = 90).

	Domain	Mean ± SD	Range	Male *n* (%)	Female *n* (%)	t
Role value	Total	7.38 ± 3.75	0.00–16.00	32 (35.6)	58 (64.4)	−0.376
K-LBI	Health	15.67 ± 7.60	2.00–45.00	32 (35.6)	58 (64.4)	1.170
	Relationship	15.00 ± 8.01	1.00–41.00			0.492
	Identity	25.77 ± 1.96	5.00–61.00			−3.086 *
	Challenge and interest	22.24 ± 9.84	2.00–46.00			−0.727
	Total	72.74 ± 28.66	23.00–159.00			24.078 **
WHOQOL-BREF	Physical Health	20.56 ± 0.89	14.00–28.00	32 (35.6)	58 (64.4)	0.141
	Psychological	15.42 ± 2.47	9.00–11.00			−1.024
	Social Relationships	9.64 ± 1.73	5.00–15.00			0.174
	Environment	29.13 ± 4.76	14.00–33.00			0.083
	Total	86.06 ± 2.947	53.00–119.00			−0.081

* *p* < 0.05, ** *p* < 0.01, K-LBI: Occupational balance; WHOQOL–BREF: Quality of life.

**Table 3:** Results of analysis of covariance for corrected quality of life according to K-LBI (*n* = 90).

	Sum Sq	Df	Mean Sq	F	*p*
Covariance (Role value)	1901.763	1	1901.763	1.284	0.285
K-LBI	570.632	3	190.211		
Error	12,596.507	85	148.194		
Sum	681,419.00	90			

**Table 4:** Correlations of participants among occupational balance, quality of life, and role value (*n* = 90).

	K-LBI ^†^				WHOQOL-BREF ^§^		
	Health ^†^	Relationship	Identity	Challenge and Interest	Physical Health	Psychological	Social Relationships
Relationship ^†^	0.942 **						
Identity ^†^	622 **	0.689 **					
Challenge and interest ^†^	0.800 **	0.896 **	0.767 **				
Physical Health ^§^	0.196	0.208 *	0.258 **	0.225 *			
Psychological ^§^	0.319 **	0.400 *	0.337 **	0.348 **	0.052 **		
Social Relationships ^§^	0.397 **	0.461 **	0.397 **	0.422 **	0.628 **	0.343 **	
Environment ^§^	0.322 **	0.353 **	0.318 **	0.355 **	0.681 **	0.169 *	0.862 **
	WHOQOL-BREF total				Role value total		
Role value total	0.343 **						
K-LBI total	0.386 **				0.323 **		

* *p* < 0.05, ** *p* < 0.01, ^†^ means K-LBI sub-items; ^§^ means WHOQOL-BREF sub-items.

**Table 5:** The effect of role value and occupational balance on quality of life (*n* = 90).

Variables	B	SE	β	T	*p*
Constant	66.952	2.306		29.029	0.000
Appearance management	2.645	0.790	0.308	3.350	0.001

R^2^ = 0.517, Adju-R^2^ = 0.251, F = 11.225, *p* = 0.001.

**Figure A1:**
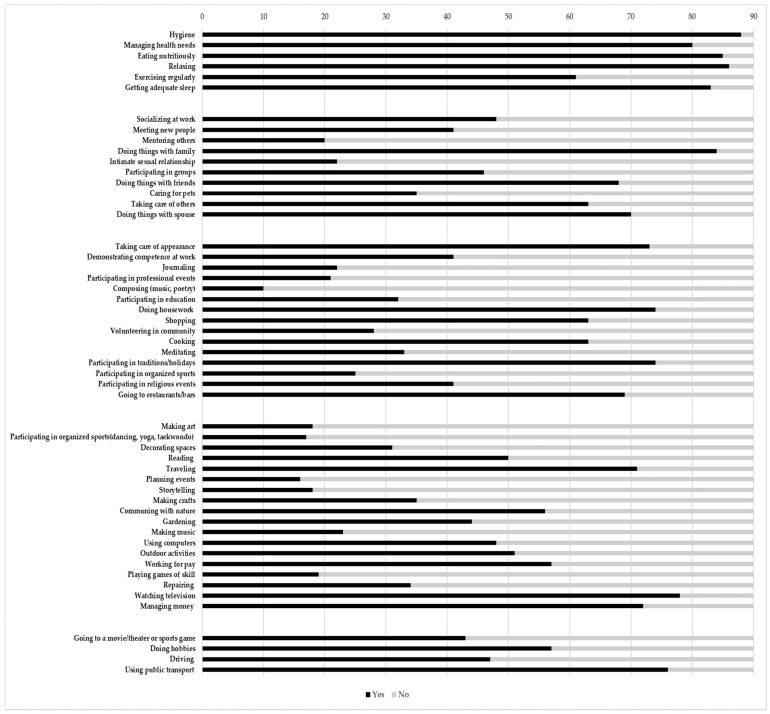
Rate of engaged occupation as measured on the K-LBI.

**Figure A2:**
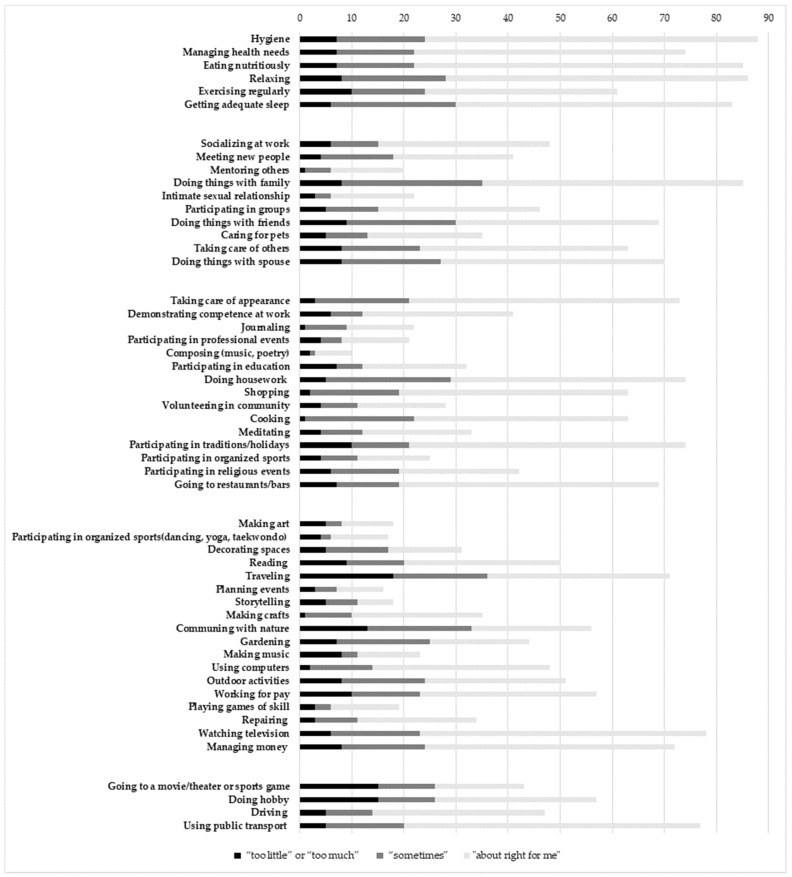
Appropriate rate of time use among occupations of participants.

## Data Availability

This is possible with the consent of the authors.
